# Practical Synthesis of Quinoline-Protected Morpholino Oligomers for Light-Triggered Regulation of Gene Function

**DOI:** 10.3390/molecules25092078

**Published:** 2020-04-29

**Authors:** Davide Deodato, Timothy M. Dore

**Affiliations:** 1 New York University Abu Dhabi, PO Box 129188, Abu Dhabi, UAE; 2 Department of Chemistry, University of Georgia, Athens, GA 30602, USA

**Keywords:** photoactivation, photo-uncaging, antisense reagents, morpholino oligomers, ccMO, click chemistry

## Abstract

Photoactivatable cyclic caged morpholino oligomers (ccMOs) represent a promising tool to selectively regulate gene expression with spatiotemporal control. Nevertheless, some challenges associated with the preparation of these reagents have limited their broader use in biological settings. We describe a novel ccMO design that overcomes many of the challenges and considerably expedites the synthetic preparation. The key factor is the introduction of an ethynyl function on the photocleavable linker to facilitate the use of a Huisgen 1,3-dipolar cycloaddition for the coupling reaction with the oligonucleotide. Compared to previous strategies, this modification reduces the number of synthetic steps and significantly improves the total yield and the stability of the linker. We used the alkynyl-functionalized linker for the preparation of two different ccMOs targeting the mRNA of the glutamic acid decarboxylase genes, *gad1* and *gad2*. HPLC analysis confirms that the caging strategy successfully inhibits the DNA binding ability, and the activity can be restored by brief illumination with 405-nm light. Overall, the straightforward preparation together with the clean and fast photochemistry make these caged antisense reagents excellent tools to modulate gene function in-vivo with spatial and temporal precision.

## 1. Introduction

Antisense reagents represent powerful tools used to selectively modify the expression of a gene of interest, avoiding the use of labor-intensive gene knockout techniques. Among the various classes of reagents developed so far, phosphorodiamidate morpholino oligomers (MOs) [1] are an interesting and successful example. Unlike other antisense reagents, MOs possess a neutral backbone made of morpholino rings interconnected through phosphorodiamidate bonds. This “unnatural” structure gives them several advantages, such as cost-effective preparation, high binding affinity, stability to nuclease degradation, and high aqueous solubility [2,3,4]. These properties often make MOs the reagent of choice for in vitro and, especially, in vivo gene knockdown experiments [5,6,7,8].

Nevertheless, the injection of MOs causes immediate and organism-wide gene silencing, precluding the use of these tools for the stage- or tissue-selective studies. This issue has been addressed with the introduction of photoactivatable (or “caged”) versions (cMOs) [9], where the MO is covalently attached to a photoremovable protecting group (PPG) that masks its activity. The activity can be restored by light exposure, which represents an ideal exogenous stimulus because it causes minimal damage to the cell or organism and is simple to use. Different strategies have been used to design efficient photoactivatable MOs. Earlier work used an MO [9,10,11] or RNA [12] complementary inhibitor sequence (containing a photocleavable moiety) to bind to the morpholino by Watson–Crick base pairing and suppress its activity. Irradiation at 360–365 nm resulted in the separation of the inhibitor sequence and exposure of the MO active sequence. Another strategy employed cMOs containing multiple nucleobases protected with a nitrobenzyl-based PPG, which were subsequently uncaged upon UV irradiation (25 W lamp) [13]. Despite the relative success of these approaches, they still suffer several limitations, including challenging and laborious synthesis, the requirement of high-power UV light for complete photolysis, and the presence of background activity from the caged sequence.

**Figure 1:**
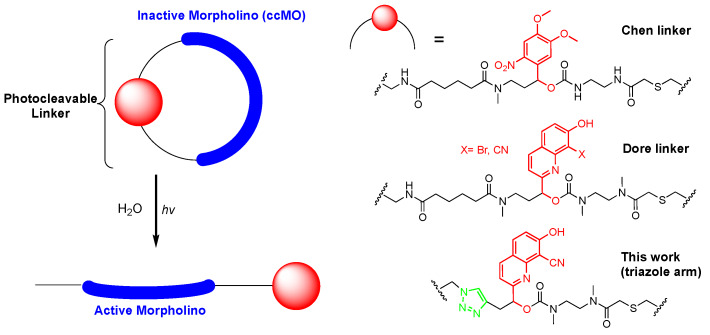
Schematic representation of the photoactivatable cyclic caged morpholino oligomers (ccMO) activation strategy. Structures of the photocleavable linkers described in the literature and the linker developed in this work. Photoremovable protecting groups (PPGs) are depicted in red.

Many of these issues were resolved with the development of cyclic caged morpholino oligomers (ccMOs) [14,15,16,17,18,19], where the oligo is cyclized using a photocleavable linker between the two ends of the sequence (Figure 1). End-to-end circular oligonucleotides have already been described [20], and photolabile versions have successfully been employed for the photoactivation of various types of oligos, including DNA [21], DNAzyme [22], miRNA [23], and siRNA [24]. In the case of MOs, it has been shown that forcing the sequence into a closed ring decreases the RNA hybridization ability by more than four-fold [15] until linearization, triggered by light exposure, restoring the active morpholino. This strategy has several advantages, such as: (1) easier synthesis, because it does not require the preparation of the complementary inhibitory sequence; (2) a simpler purification step since the circular MO is easily separated by HPLC from the unreacted linear sequences; (3) the absence of off-target effects, because the protecting strategy relies on covalent modification instead of Watson–Crick base pairing. As a result, various ccMOs have been developed and successfully used to regulate gene function with spatiotemporal precision. The Chen and Tang labs independently designed the first examples of ccMOs and successfully applied the strategy for selective control of gene function in zebrafish embryos [14,15]. Sequential gene knockdown was achieved by employing wavelength-selective ccMOs, in which two orthogonal PPGs (nitrobenzyl- and coumarin-based) were used to cage different sequences [16]. Dmochowski et al. developed ruthenium-caged circular MOs targeting the mRNA of the *chordin* and *notail* zebrafish genes, achieving efficient gene silencing with 450-nm irradiation [18].

Inspired by such work, we designed a ccMO using the highly efficient 8-bromo and 8-cyano-7-hydroxyquinolinyl-methyl (BHQ and CyHQ) PPGs and applied the strategy to the photoactivation of MO sequences targeting the mRNA of the glutamic acid decarboxylase genes (*gad1* and *gad2*) of zebrafish [19]. We successfully employed these tools to bypass glutamic acid decarboxylase (GAD)-induced morphological defects and study the effects of GABAergic transmission during brain development. The main advantages of these ccMOs were their stability toward spontaneous and enzyme-catalyzed degradation and the possibility to carry out the photolysis reaction with ambient light, due to the favorable properties of the PPGs [25]. One disadvantage was that the synthesis of the linker moiety, inspired by Chen’s work [14], required multiple moderate-to-low yielding steps, which limited the practicality of these ccMOs.

In this work, we introduced a novel cyclic caged MO with a different linker, which significantly expedited the synthetic preparation while retaining all the other excellent properties, including a fast and clean photolysis reaction with complete restoration of the oligonucleotide binding ability.

## 2. Results

### 2.1. Design and Synthesis of the Linker Moiety

The previous linkers reported by Chen [14] and Dore [19], consisted of a photocleavable unit (nitrobenzyl- or quinoline-based) bearing two arms with selected functionalities in order to be connected to the 5′ and 3′ ends of the MO sequence (Figure 1). The synthesis of these molecules (starting from the benzaldehyde precursor) required a sequence of 11 steps for Chen’s and nine steps for Dore’s linker (CyHQ case), with total yields of 8% and 5%, respectively. The challenging and laborious nature of their syntheses is one of the main reasons behind the sparse application of ccMOs in biological studies.

**Scheme 1:**
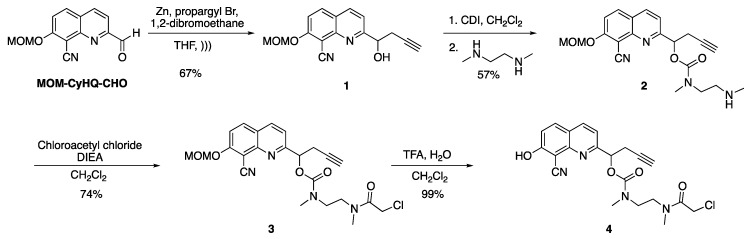
Synthesis of linker **4** starting from **MOM-CyHQ-CHO** [27].

With the aim of facilitating the synthetic preparation, we designed a new CyHQ-based linker (compound **4**, Scheme 1) bearing a simple propargyl unit on one of the arms, which can be connected to an azide-functionalized MO via Huisgen 1,3-dipolar cycloaddition, a click chemistry reaction. This strategy reduced the overall number of steps by avoiding the low-yielding preparation of the adipoyl amide arm (Figure 1, Chen and Dore linker) and improved the efficacy of MO coupling by exploiting the Huisgen 1,3-dipolar cycloaddition, a simple, efficient and water-favorable “click” chemistry reaction [26]. The synthetic pathway leading to linker **4** starts from benzaldehyde **MOM-CyHQ-CHO** (Scheme 1), obtained in multi-gram quantities according to the method previously reported [27]. The propargyl appendage was installed via a Barbier reaction using 1,2-dibromoethane as the activator under ultrasound stirring, affording secondary alcohol **1**. The second arm of the linker was prepared by coupling the activated imidazolyl carbamate, generated after reacting **1** with 1,1′-carbonidiimidazole, with N N’-dimethylethylenediamine, furnishing compound **2** in good yield (57% over two steps). The latter was reacted with chloroacetyl chloride obtaining α-activated amine **3**, which was deprotected from the acid-labile MOM group with trifluoroacetic acid in CH_2_Cl_2_, furnishing final compound **4**.

The total yield for the preparation of the new linker was 28% over five steps, representing a 5.6-fold improvement from the previous methodology (Dore’s linker). In addition, the stability of the final linker is improved by substituting the moisture-sensitive succinimidyl ester present in Chen’s and Dore’s linkers, with the terminal alkyne. As a result, linker **4** is bench-stable (if kept in the dark), whereas the previous versions require storage at −30 °C under an inert atmosphere.

### 2.2. MO Coupling and Final Cyclization

**Scheme 2:**
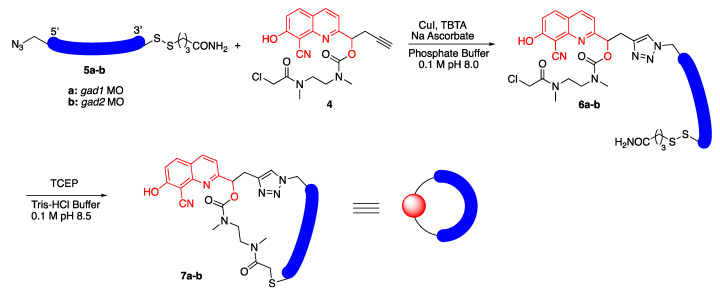
Synthesis of ccMOs **7a**–**b**.

As a proof of concept, we selected the same MO sequences used in our previous work [19] (targeting the mRNA of the *gad1* and *gad2* genes), to be photocaged with the new linker **4**. In this case, the linear MO (**5a**–**b**, Scheme 2) contained an azide functionality on the 5′ end, to be coupled to the linker through a click chemistry reaction, and a disulfide amide modification on the 3′ end for the ultimate macrocyclization.

Final ccMOs **7a**–**b** were prepared according to the two-step procedure given in Scheme 2. The coupling reaction between **4** and **5a**–**b** was achieved via Huisgen 1,3-dipolar azide-alkyne cycloaddition, using copper iodide as a catalyst in phosphate buffer. Tris[(1-benzyl-1*H*-1,2,3-triazol-4-yl)methyl]amine (TBTA) was used as a Cu(I) stabilizer and sodium ascorbate as a reducing agent. Under these conditions, the reaction was completed within two hours after shaking the reaction mixture with a vortex at room temperature in the dark. The final macrocyclization was achieved by the treatment of **6a**–**b** with a freshly prepared solution of tris(2-carboxyethyl)phosphine hydrochloride in Tris buffer. The in-situ generated sulfhydryl performed a nucleophilic substitution on the activated α-carbonyl, affording the ccMOs **7a-b** in moderate yields (25–42% over two steps, determined by UV analysis). Purification was carried out by size exclusion chromatography (on a NAP-5 column, eluting with water), and the final compounds were characterized by LC-MS analysis (see the Materials and Methods). The full structures of **7a**–**b** are given in the Supplementary Material (Figure S1).

**Figure 2:**
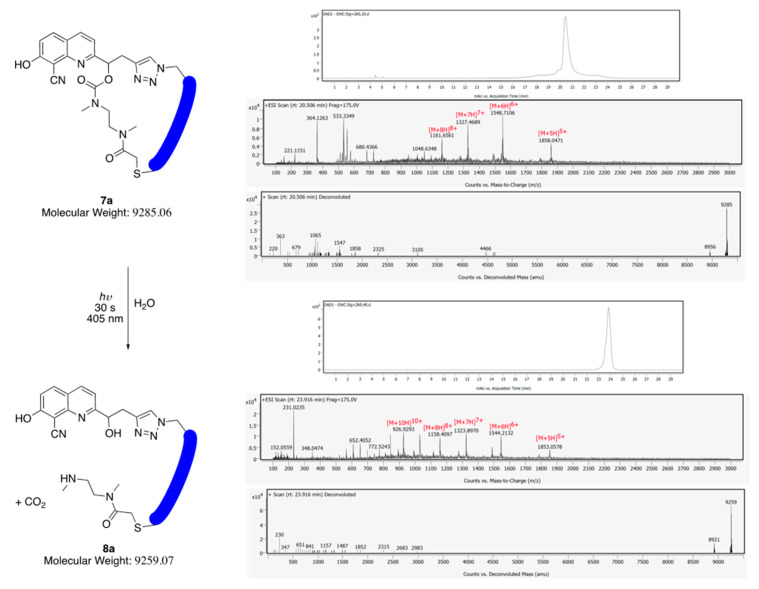
Photolysis reaction of **7a** under UV illumination (405 nm, LED) in water monitored by LC-MS. Chromatograms, electrospray ionization high-resolution mass (ESI-HRMS) traces and deconvoluted spectra are given before (top) and after (bottom) photolysis.

The photolysis reaction was investigated by irradiating aqueous solutions of **7a** with 405-nm light for 30 s using an LED source, monitoring the reaction by LC-MS before and after irradiation, using an electrospray ionization (ESI) source (Figure 2). The mechanism of the photolysis reaction of quinoline-based PPGs is well known [28] and proceeds with the generation, after excited-state C-O bond cleavage, of a benzylic carbocation that is rapidly quenched by the solvent, resulting in a hydroxy species. In the case of a carbamate derivative like **7a** [19,29], an additional decarboxylation reaction occurred with the formation of the corresponding amine **8a** (Figure 2). The net molecular weight loss of the process was 26 *m*/*z*, as confirmed by the MS analysis. The MS trace of **7a** before UV irradiation (Figure 2, top panel) showed peaks corresponding to multi-charge ionization states of the MO, and the deconvoluted spectrum confirmed the molecular weight of **7a**. Note that the small peak identified at 8956 *m*/*z* corresponds to a ccMO missing one nucleotide, an impurity also found in the commercial starting material **5a**. After irradiation, a new set of multi-charged species appeared (Figure 2, bottom panel), which gave, after deconvolution, the expected molecular weight of the photolysis product **8a** (9259 *m*/*z*). This result confirmed that the photolysis reaction of the ccMO proceeds rapidly and efficiently since no other photolysis by-products were detected.

### 2.3. In-vitro DNA Hybridization

**Figure 3:**
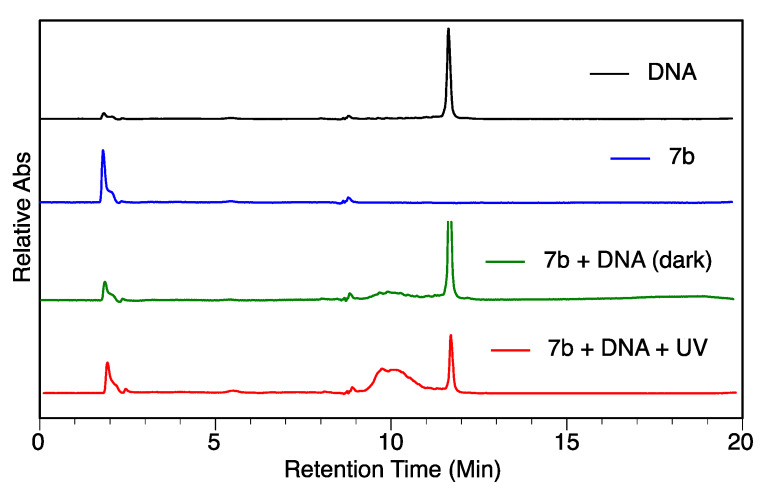
HPLC traces for the DNA hybridization experiments upon photoactivation. The complementary ssDNA sequence to **7b** was used (black line). The ratio of ssDNA to MO used was 2:1. A broad peak (*t*_R_ = 9.5–10.5, AUC = 526) corresponding to the duplex between linearized **7b** and its complementary ssDNA appeared after UV irradiation (red line). Without irradiation (green line) only minor hybridization was detectable (*t*_R_ = 9.5–10.5, AUC = 90).

The crucial property of a successful ccMO is the effective restoration of oligo-hybridization capability upon photoactivation. MOs are typically used to block a complementary sequence of RNA (mRNA, maturation of miRNA, or splicing of pre-mRNA), but they can form a stable duplex with single-stranded DNA (ssDNA) as well [30,31]. We investigated the ability of **7b** to bind to a 25-mer complementary oligodeoxynucleotide (before and after UV irradiation) using HPLC analysis, following a similar experimental setup to that previously reported [15]. Briefly, separations were carried out using ion-exchange chromatography, and solutions of **7b** (kept in the dark or irradiated with a 405-nm LED) were incubated with the complementary ssDNA at 40 °C for 30 min before injection into the HPLC instrument (Figure 3).

Under these experimental conditions, the negatively charged ssDNA (black trace) moved slowly through the ion-exchange column (*t*_R_ = 11.9 min), whereas the neutral ccMO (blue trace) eluted quickly (*t*_R_ = 2.0 min). Note that under these conditions, it is not possible to chromatographically separate the caged and photoactivated MO since, being both uncharged, they do not interact with the ion-exchange column and elute at the same retention time (2.0 min). When injecting a mixture of ssDNA and photoactivated **7b** (red trace), a new broad peak appeared (*t*_R_ = 9.5–10.5 min), corresponding to the duplex between the two species. Without prior photoactivation of **7b** (green trace), only minimal hybridization was observed (a 5.8-fold decrease of the area under the curve (AUC), see Figure 3 caption), due to the residual binding affinity of the ccMO. An analogous result (a 4.1-fold increase from baseline activity) was obtained by Wang and coworkers from the photo-linearization of a circular MO-*ntl* [15]. Additionally, it has been shown that the basal activity of the ccMO only moderately impacts the phenotypical effects in a real biological setup, whereas dramatic outcomes are observed upon linearization [14].

Taken together, the results from the DNA hybridization experiments highlight the potential for the ccMO to be successfully used for the light-triggered modulation of gene expression.

## 3. Discussion

Manipulating the expression of a gene of interest is a useful approach to study biological processes, and achieving spatiotemporal control over gene knockdown is often required. In this context, photoactivatable antisense reagents, and in particular ccMOs, represent attractive tools compared to other optogenetic approaches [32,33,34], because of their modularity, efficacy, and simplicity of use in biological preparations. Despite their excellent properties, since their introduction in 2012 [14,15], only a few other works have employed ccMOs in in-vivo experiments [16,17,18,19]. We speculate that one of the reasons behind the scarcity of use is related to difficult synthetic preparation.

To overcome these challenges, we designed a ccMO with a key modification (a 1,2,3-triazole ring) in the linker connecting the two ends of the MO. As a result, we synthesized a new photocleavable linker (**4**) bearing an ethynyl group to be connected to a 5′-azido MO via a click chemistry reaction. The synthetic preparation of this quinoline derivative has several advantages in comparison to our previous method, including a reduced number of steps (5 vs. 11), an improved final yield (28% vs. 5%), and increased stability, enabling it to be stored at room temperature while the previous versions (including Chen’s) required cold (−30 °C) and moisture-free conditions. In addition, the quinoline-based PPG has superior photochemical properties (faster photolysis kinetics, higher quantum yield, and red-shifted lambda max) compared to the nitroveratryl-based PPG in Chen’s linker [25,35]. Compound **4** was effectively conjugated to the 5′-azido-3′-disulfide-MOs **5a** and **5b** (encoding for the mRNA of the zebrafish genes *gad1* and *gad2*, respectively) through Huisgen 1,3-dipolar azide-alkyne cycloaddition. These new coupling conditions enabled us to generate intermediate linear MOs **6a**–**b** faster (reaction time = 1–2 h) and in higher purity since most of the reagents and by-products crashed out of the solution and were easily removed by centrifugation. We also optimized the final macrocyclization by using a freshly prepared solution of TCEP (as a disulfide-reducing agent) instead of the resin-bound version used in the previous methods, which requires multiple swelling steps to ensure homogeneous activation. LC-MS was used for the characterization of the final ccMOs and to investigate the products of the photolysis reaction in water (Figure 2). The MS peaks identified corresponded to the multi-ionic states of the ccMO and the expected linear photoproduct **8a**, resulting from the light-triggered C-O bond cleavage and subsequent decarboxylation.

We confirmed that the oligonucleotide binding ability is effectively blocked by the caging strategy and can be promptly restored by illumination, a key property for a photoactivatable construct. The duplex formation between an MO and its complementary ssDNA can be detected by ion-exchange chromatography [15], and we applied the same method to study the ability of our ccMO to hybridize a complementary ssDNA sequence. The minimal duplex formation was detected between the caged morpholino **7b** and its complementary oligodeoxynucleotide (Figure 3, green trace), whereas prior exposure of the solution of **7b** to UV irradiation resulted in a 5.8-fold increase of the MO-ssDNA duplex peak (Figure 3, red trace).

In summary, we described the design and synthesis of photoactivatable MOs to be used for the selective in-vivo photoregulation of gene expression. Compared to previous examples, our ccMOs are easier to prepare and can be designed to target any gene of interest, making the strategy broadly applicable. We believe that these tools will be useful to investigate the function of developmentally important genes, where it is essential to achieve spatiotemporal control over gene knockdown.

## 4. Materials and Methods

### 4.1. General Information

All reagents and solvents were purchased from commercial sources and used without further purification. **MOM-CyHQ-CHO** was prepared as previously described [27]. All reactions were carried out using glassware except for the MO couplings, which were performed in microcentrifuge tubes. Anhydrous reactions were performed at a positive pressure of dry nitrogen gas. Anhydrous THF was obtained by distillation over Na/benzophenone. Purifications were carried out using flash chromatography on an Isolera Spektra 4 with Biotage SNAP cartridges packed with KP-Sil silica. ^1^H NMR and ^13^C NMR spectra were recorded using a Bruker Avance™ III HD 500 MHz spectrometer (Billerica, MA, USA). UV spectra for MO quantification were recorded on a Nanodrop 2000c. HPLC was performed on an Agilent Infinity series system with an autosampler and diode array detector. Separations were performed using Zorbax eclipse C-18 reverse phase columns 150 × 4.6 mm with 8 μm particle size and gradient elution at a flow rate of 0.5 mL min^−1^: eluent A was acetonitrile and eluent B was water. The analysis started with 2% of eluent A, which was linearly increased up to 100% over 20 min, and then kept at 100% for 5 min. HRMS/LC-MS was performed on an Agilent 6540 HD Accurate Mass QTOF/LC/MS (Santa Clara, CA, USA) with electrospray ionization (ESI) using a fragmentor voltage of 175 V.

### 4.2. Preparation of Linker 4

*2-(1-hydroxybut-3-yn-1-yl)-7-(methoxymethoxy)quinoline-8-carbonitrile* (**1**). Zinc powder was activated by repeated washing with 0.5 M HCl, followed by H_2_O and EtOH and dried at 120 °C for 12 h prior to use. After activation, Zn (107 mg, 1.65 mmol, 4 eq) and 1,2-dibromoethane (0.02 mL, 0.21 mmol, 0.5 eq) were suspended in anhydrous tetrahydrofuran (THF) (5 mL) and sonicated with an ultrasonic bath for 10 min under nitrogen atmosphere. Propargyl bromide (0.09 mL, 0.83 mmol, 2 eq) was slowly added and the mixture was sonicated for a further 10 min. A solution of **MOM-CyHQ-CHO** (100 mg, 0.41 mmol, 1 eq) in anhydrous THF (2 mL) was added and the mixture was sonicated for 1 h under nitrogen atmosphere. After completion, the reaction was diluted with EtOAc (30 mL) and filtered over Celite. The filtrate was washed successively with H_2_O (2 × 30 mL) and brine (2 × 30 mL), dried over MgSO_4_, and concentrated to dryness. The crude product was purified by column chromatography (hexanes/EtOAc gradient) to yield **1** (78 mg, 0.27 mmol, 67%). ^1^H NMR (500 MHz, DMSO-*d_6_,* δ) 8.47 (d, *J* = 8.4 Hz, 1H), 8.29 (d, *J* = 8.4 Hz, 1H), 7.76 (d, *J* = 8.5 Hz, 1H), 7.69 (d, *J* = 8.5 Hz, 1H), 6.01 (d, *J* = 8.4 Hz, 1H), 5.61–5.52 (m, 2H), 4.94 (t, *J* = 5.8 Hz, 1H, O-H), 3.49 (s, 3H), 2.91–2.82 (m, 1H), 2.71 (s, 1H), 2.68 (m, 1H); ^13^C NMR (126 MHz, chloroform-*d*, δ): 163.22, 162.28, 147.16, 137.01, 133.64, 122.91, 118.57, 115.74, 114.29, 99.46, 95.10, 93.63, 80.15, 71.30, 56.95, 29.71, 28.24 (diastereotopic CH_2_); HRMS (ESI-QTOF) *m*/*z*: [M + H]^+^ calcd for C_16_H_14_N_2_O_3_, 283.1077; found, 283.1035.

*1-(8-cyano-7-(methoxymethoxy)quinolin-2-yl)but-3-yn-1-yl-methyl(2-(methylamino)ethyl)carbamate* (**2**). 1,1′-Carbonyldiimidazole (CDI) (240 mg, 1.49 mmol, 7 eq) was dissolved in CH_2_Cl_2_ (6 mL) and the solution was placed into an ice-bath. A solution of **1** (60 mg, 0.21 mmol, 1 eq) in CH_2_Cl_2_ (4 mL) was added dropwise. The mixture was stirred at 0 °C for 1 h and then allowed to reach room temperature and stirred until complete consumption of starting material (30 min, monitored by LC/MS). The reaction was quenched with H_2_O (20 mL), the organic layer was separated and the aqueous layer was extracted with CH_2_Cl_2_ (2 × 5 mL). The organic phases were combined in a round-bottomed flask, cooled by placing the flask into an ice-bath and *N*,*N*’-dimethylethylenediamine (1.1 mL) was added dropwise. The resulting mixture was stirred at 0 °C for 2 h, and then at room temperature for 1 h. The reaction was then concentrated to dryness and the crude product was purified by column chromatography (CH_2_Cl_2_/MeOH gradient) to yield **2** (48 mg, 0.12 mmol, 57%). ^1^H NMR (500 MHz, chloroform-*d*, δ) 8.15 (d, *J* = 8.4 Hz, 1H), 7.97 (d, *J* = 9.2 Hz, 1H), 7.54 (m, 2H), 6.05 (t, *J* = 6.0 Hz, 1H), 5.45 (s, 2H), 3.64 (s, 1H, N-H), 3.58 (s, 3H), 3.56–3.47 (m, 3H), 3.15–3.11 (m, 2H), 2.99 (m, 2H), 2.92 (m, 1H), 2.87 (q, *J* = 6.3 Hz, 1H), 2.51 (d, *J* = 31.9 Hz, 3H), 1.97 (d, *J* = 12.3 Hz, 1H). ^13^C NMR (126 MHz, chloroform-*d*, δ) (rotamers) 162.19, 161.47, 155.95 (155.42), 148.10, 136.77, 133.63, 122.82, 119.08 (118.83), 115.74, 114.68, 99.64, 95.06, 79.86, 75.02, 70.76 (70.67), 56.88, 54.92 (54.01), 48.97 (48.36), 35.81 (35.73), 35.38 (34.89), 24.79. HRMS (ESI-QTOF) *m*/*z*: [M + H]^+^ calcd for C_21_H_24_N_4_O_4_, 397.1870; found, 397.1818.

*1-(8-cyano-7-(methoxymethoxy)quinolin-2-yl)but-3-yn-1-yl-(2-(2-chloro-N-methylacetamido)ethyl)(methyl)carbamate* (**3**). To a solution of **2** (48 mg, 0.12 mmol, 1 eq) in CH_2_Cl_2_ (5 mL), diisopropylethylamine (0.04 mL, 0.24 mmol, 2 eq) was added dropwise and the mixture was cooled by placing the flask into an ice-bath. A freshly prepared (by treatment of chloroacetic acid with thionyl chloride in CH_2_Cl_2_ at 0 °C) 1 M solution of chloroacetylchloride in CH_2_Cl_2_ (0.24 mL, 0.24 mmol, 2 eq) was added dropwise and the resulting mixture was stirred at 0 °C for 2 h, and then at room temperature for 1 h. The reaction was quenched with H_2_O (20 mL), extracted with CH_2_Cl_2_ (3 × 20 mL), dried over MgSO_4_, and concentrated to dryness. The crude product was purified by column chromatography (hexanes/EtOAc gradient) to yield **3** (41 mg, 0.09 mmol, 74%). ^1^H NMR (500 MHz, chloroform-*d*, δ) 8.18 (d, *J* = 8.5 Hz, 1H), 7.99 (d, *J* = 9.2 Hz, 1H), 7.55 (dd, *J* = 9.4, 6.7 Hz, 2H), 6.11–5.98 (t, *J* = 9.8 Hz, 1H), 5.46 (s, 2H), 4.27–3.94 (m, 2H), 3.77–3.63 (m, 2H), 3.59 (s, 3H), 3.57–3.41 (m, 1H), 3.28–3.03 (m, 7H), 2.99 (d, *J* = 5.8 Hz, 2H), 2.09–1.93 (m, 1H). ^13^C NMR (126 MHz, chloroform-*d*, δ) (rotamers) 166.78 (166.71), 162.27 (162.17), 161.70 (161.12), 155.76 (155.27), 148.27 (148.08), 136.79 (136.73), 133.63 (133.56), 122.95, 119.78 (118.50), 115.89 (115.73), 114.55, 99.83 (99.75), 95.07, 79.90 (79.61), 75.15, 70.76 (70.62), 56.90, 48.63, 46.79 (46.28, 46.20, 45.77), 41.52 (41.45, 41.00), 36.64 (35.98, 35.94), 35.55 (34.87), 24.88 (24.69). HRMS (ESI-QTOF) *m*/*z*: [M + H]^+^ calcd for C_23_H_25_ClN_4_O_5_, 473.1586; found, 473.1539.

*1-(8-cyano-7-hydroxyquinolin-2-yl)but-3-yn-1-yl-(2-(2-chloro-N-methylacetamido)ethyl)(methyl)carbamate* (**4**). To a solution of **3** (30 mg, 0.06 mmol, 1 eq) in CH_2_Cl_2_ (2 mL), H_2_O (0.1 mL) and trifluoroacetic acid (0.4 mL) were added. The mixture was stirred at room temperature in the dark for 3 h. After reaction completion (monitored by LC/MS) the mixture was concentrated to dryness and triturated multiple times with THF, affording pure linker **4** (26 mg, 0.06 mmol, 99% yield). ^1^H NMR (500 MHz, chloroform-*d*, δ) 8.24 (d, *J* = 8.4 Hz, 0.5H, rotamer), 8.17 (d, *J* = 5.0 Hz, 0.5H, rotamer), 7.81 (dd, *J* = 18.4, 9.0 Hz, 1H), 7.37 (d, *J* = 8.3 Hz, 1H), 7.07 (d, *J* = 9.0 Hz, 0.5H, rotamer), 7.01 (d, *J* = 9.0 Hz, 0.5H, rotamer), 6.01 (bs, *J* = 6.4 Hz, 1H, OH), 5.94 (dd, *J* = 7.1, 4.5 Hz, 1H), 4.33–4.25 (m, 1H, rotamer), 4.17 (d, *J* = 4.4 Hz, 1H, rotamer), 4.01–3.86 (m, 2H), 3.79–3.62 (m, 2H), 3.28 (m, 3H), 3.13–3.08 (m, 3H), 3.05–2.98 (m, 1H, rotamer), 2.92 (ddd, *J* = 17.1, 7.1, 2.6 Hz, 1H, rotamer), 2.03 (m, 1H). ^13^C NMR (126 MHz, chloroform-*d*, δ) (rotamers) 168.54, 163.25, 160.42, 156.99, 147.08, 138.12 (137.86), 134.05 (133.88), 121.80, 118.62 (118.47), 116.20, 94.26 (93.88), 79.47 (78.97), 75.94, 71.17 (71.10), 46.47 (46.30), 46.09, 41.58 (40.85), 36.81 (36.00), 35.87 (34.84), 29.70, 25.36 (25.14). HRMS (ESI-QTOF) *m*/*z*: [M + H]^+^ calcd for C_21_H_21_ClN_4_O_4_, 429.1324; found, 429.1312.

### 4.3. Preparation of ccMOs 7a–b

*CyHQ-MO*-*gad1* (**6a**) and *CyHQ-MO*-*gad2* (**6b**). The custom 25-mer MO containing 5′-azide and 3′-disulfide amide modifications (**5a** 5′-AAGGTGCAGAAGACGCCATCAGTCC-3′ or **5b** 5′-GAAACCAAAACCCGTGTGATGCCAT-3′) was obtained from Gene Tools LLC (Philomath, OR, USA) and dissolved in H_2_O to a final concentration of 1 mM. The stock solution was sampled (0.1 mL, 100 nmol, 1 eq), lyophilized in an Eppendorf tube, and re-dissolved in 0.4 mL of phosphate buffer (0.1 M, pH 8.0). A 50 mM DMSO solution of the photocleavable linker **4** (0.02 mL, 1 µmol, 10 eq) was added to the tube followed by solutions of sodium ascorbate (0.02 mL from a 50 mM stock in H_2_O, 1 µmol, 10 eq), copper (I) iodide (0.02 mL from a freshly prepared 50 mM stock in DMSO, 1 µmol, 10 eq), and tris((1-benzyl-4-triazolyl)methyl)amine (TBTA) (0.02 mL from a 50 mM stock in DMSO, 1 µmol, 10 eq). All operations, including the following one, were carried out in a dark room. The mixture was sonicated in an ultrasonic bath for 2 min and shaken on a vortexer until the reaction completed (indicated by LC/MS analysis, 1–2 h). The precipitate obtained was removed by centrifugation, and the resulting solution was purified on a NAP-5 gel-filtration column (eluting with 2 mL of Milli-Q water) and lyophilized. The resulting white solid was dissolved in H_2_O (200 µL). Acetic acid (2 µL) was added to the solution, which was then washed with chloroform (3 × 200 µL) and EtOAc (2 × 200 µL), and then neutralized with 10% aqueous NH_4_OH (20 µL). The solution was lyophilized to dryness, affording the conjugated MO product **6a** or **b** as a white solid, which was taken directly to the next step without further purification. **6a** HRMS (ESI-QTOF) *m*/*z*: [M]^+^ calcd for C_338_H_515_N_167_O_103_S_2_P_25_Cl, 9439.7029; found (after deconvolution), 9439.3209. **6b** HRMS (ESI-QTOF) m/z: [M]^+^ calcd for C_338_H_515_N_164_O_103_S_2_P_25_Cl, 9397.6828; found (after deconvolution), 9397.2859.

*CyHQ-ccMO*-*gad1* (**7a**) and *CyHQ-ccMO*-*gad2* (**7b**). **6a** or **b** was dissolved in (0.4 mL) of Tris-HCl buffer (0.1 M, pH 8.5) inside an Eppendorf tube. A fresh 15 mM solution of tris(2-carboxyethyl)phosphine (TCEP) (40 µL, 600 nmol, 6 eq) in Tris-HCl buffer was added dropwise, and the mixture was shaken on an vortexer for 12 h. All operations, including the following one, were carried out in a dark room. The mixture was purified on a NAP-5 gel-filtration column (eluting with 2 mL of H_2_O) and lyophilized, affording pure final compound as a white solid. **7a** (25 nmol, 25% yield over two steps). HRMS (ESI-QTOF) *m*/*z*: [M]^+^ calcd for C_334_H_507_N_166_O_102_SP_25_, 9285.0646; found (after deconvolution), 9285.5066. **7b** (42 nmol, 42% yield over two steps). HRMS (ESI-QTOF) *m*/*z*: [M]^+^ calcd for C_334_H_507_N_163_O_102_SP_25_, 9244.0525; found (after deconvolution), 9244.1692.

### 4.4. DNA Hybridization Experiment

All solutions were prepared in 20 mM phosphate buffer (pH 7.2) at a final concentration of 0.05 mM. A 25-mer oligodeoxynucleotide (5′-CTTTGGTTTTGGGCACACTACGGTA-3′) complementary to **7b** (Bioneer Pacific) was used in a 2:1 ratio of ssDNA to MO. Photolysis reactions were performed by irradiating the solution of ccMO for 30 s with an LED lamp (Cairn OptoLED Lite) at a wavelength of 405 nm with an average power of 12 mW. Mixtures of the ccMO and ssDNA were incubated in the dark at 40 °C for 30 min before HPLC injection. Separations were obtained by ion-exchange chromatography using a DNAPac RP (ThermoFisher) column with gradient elution at a flow rate of 0.6 mL min^−1^: eluent A was 20 mM phosphate buffer (pH 7.2), and eluent B consisted of eluent A + 2 M NaCl. The analysis started with 100% of eluent A for 4 min, then B was linearly increased up to 90% over 11 min, and then remained at 90% for 2 min. The column was then returned to 100% A over 3 min. Traces were obtained at a wavelength of 280 nm.

## Supplementary Materials

The following are available online, ^1^HNMR and ^13^C spectra of compounds **1**–**4**; HMRS spectra of compounds **6a**–**b** and **7a**–**b**.
