# Supplementary material for: Practical Synthesis of Quinoline-Protected Morpholino Oligomers for Light-Triggered Regulation of Gene Function
Source: Molecules. 2020 Apr 29;25(9):2078. doi: 10.3390/molecules25092078 (PMC7248704; doi:10.3390/molecules25092078)

molecules

Supporting Information for

Practical Synthesis of Quinoline-Caged Morpholino Oligomers for the Light-Triggered Regulation of Gene Function

Davide Deodato [1,*] and Timothy M. Dore [1,2,*]

[1] New York University Abu Dhabi, PO Box 129188, Abu Dhabi, United Arab Emirates
[2] Department of Chemistry, University of Georgia, Athens, GA 30602, USA
* Correspondence: davide.deodato@nyu.edu; timothy.dore@nyu.edu

Table of Contents

FIGURE S1. STRUCTURES OF COMPOUNDS 7A AND 7B. 3

^{1}H AND ^{13}C NMR SPECTRA 4

2-(1-hydroxybut-3-yn-1-yl)-7-(methoxymethoxy)quinoline-8-carbonitrile (**1**). 4

1-(8-cyano-7-(methoxymethoxy)quinolin-2-yl)but-3-yn-1-yl-methyl(2-(methylamino)ethyl)carbamate (**2**). 5

1-(8-cyano-7-(methoxymethoxy)quinolin-2-yl)but-3-yn-1-yl-(2-(2-chloro-N-methylacetamido)ethyl)(methyl)carbamate (**3**). 6

1-(8-cyano-7-hydroxyquinolin-2-yl)but-3-yn-1-yl-(2-(2-chloro-N-methylacetamido)ethyl)(methyl)carbamate (**4**). 7

HRMS SPECTRA... 8

CyHQ-MO-gad1 (**6a**) 8

CyHQ-MO-gad2 (**6b**) 9

CyHQ-ccMO-gad1 (**7a**).. 10

CyHQ-ccMO-gad2 (**7b**) 11

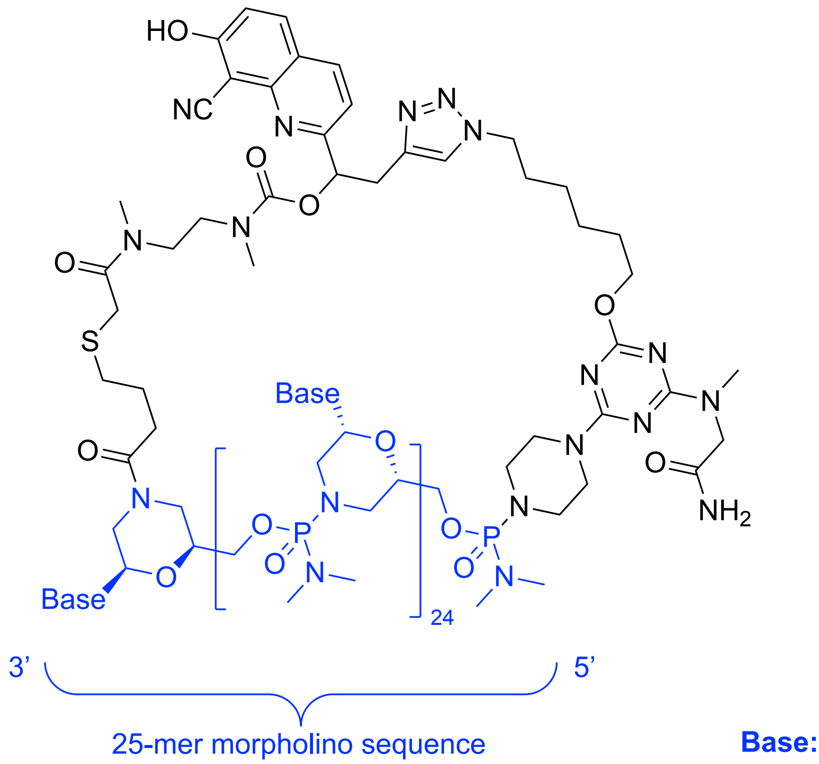

7a: 5′-AAGGTGCAGAAGACGCCATCAGTCC-3′

7b: 5′-GAAACCAAAACCCGTGTGATGCCAT-3′

Base:
A = Adenine
G = Guanine
T = Thymine
C = Cytosine

Figure S1. Full structures of compounds **7a** and **7b**.

^{1}H and ^{13}C NMR Spectra

*2-(1-hydroxybut-3-yn-1-yl)-7-(methoxymethoxy)quinoline-8-carbonitrile (**1**).*

^{1}H NMR (500 MHz, DMSO-d_6)

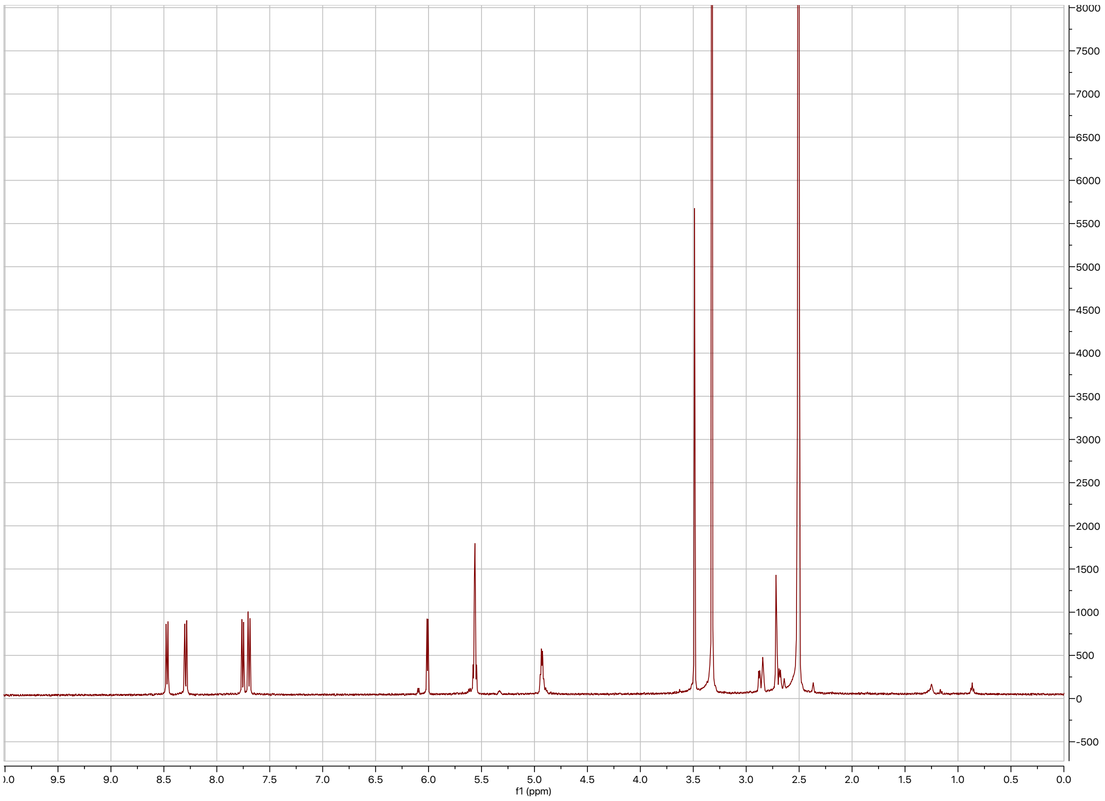

^{13}C NMR (126 MHz, chloroform-d)

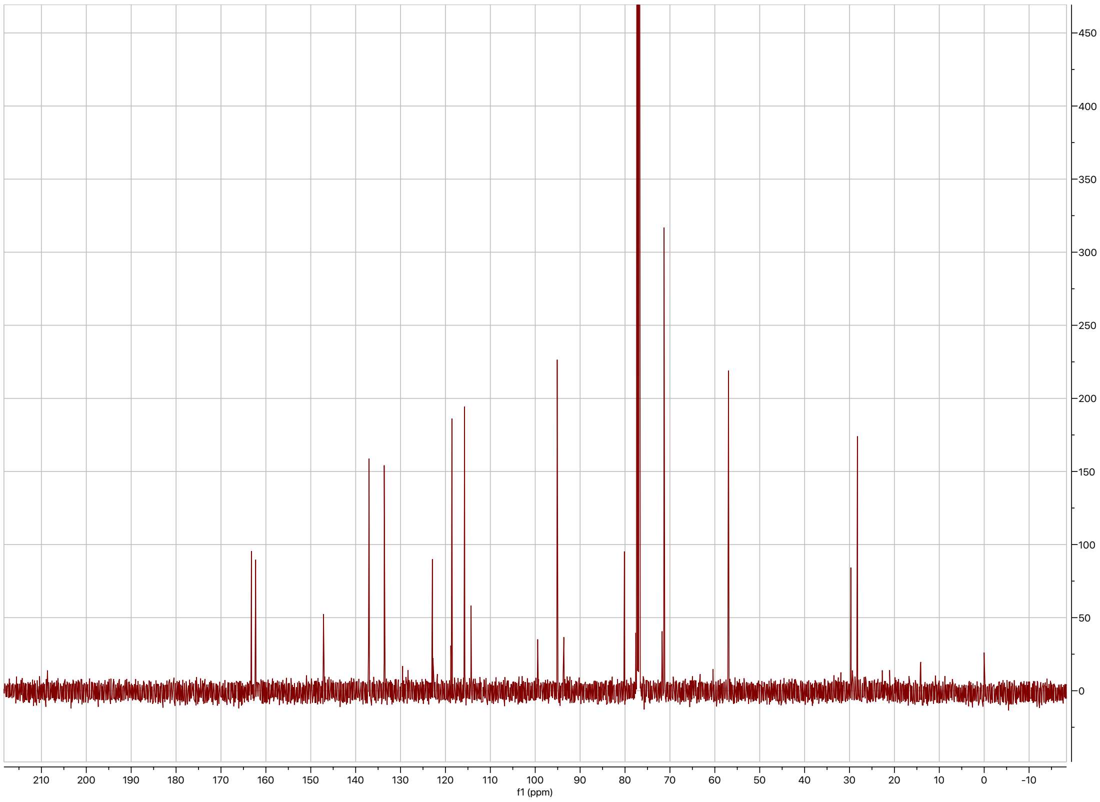

*1-(8-cyano-7-(methoxymethoxy)quinolin-2-yl)but-3-yn-1-yl-methyl(2-(methylamino)ethyl)carbamate (**2**).*

^{1}H NMR (500 MHz, chloroform-*d*)

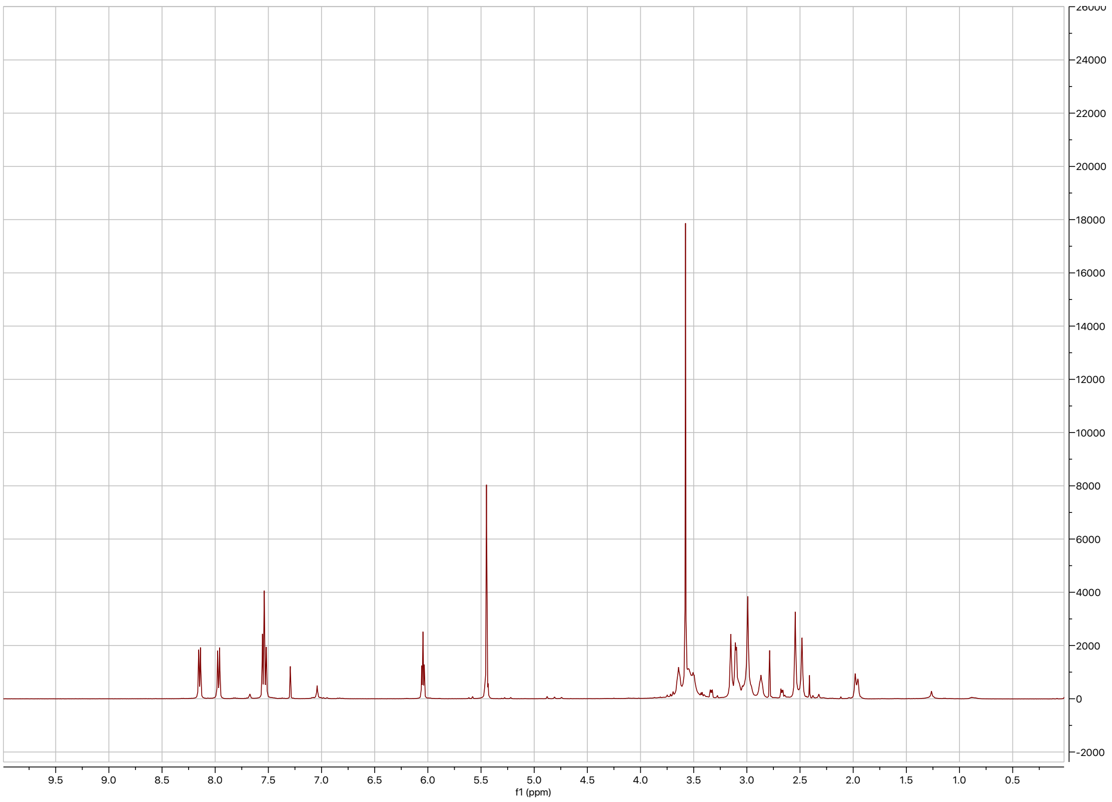

^{13}C NMR (126 MHz, chloroform-*d*)

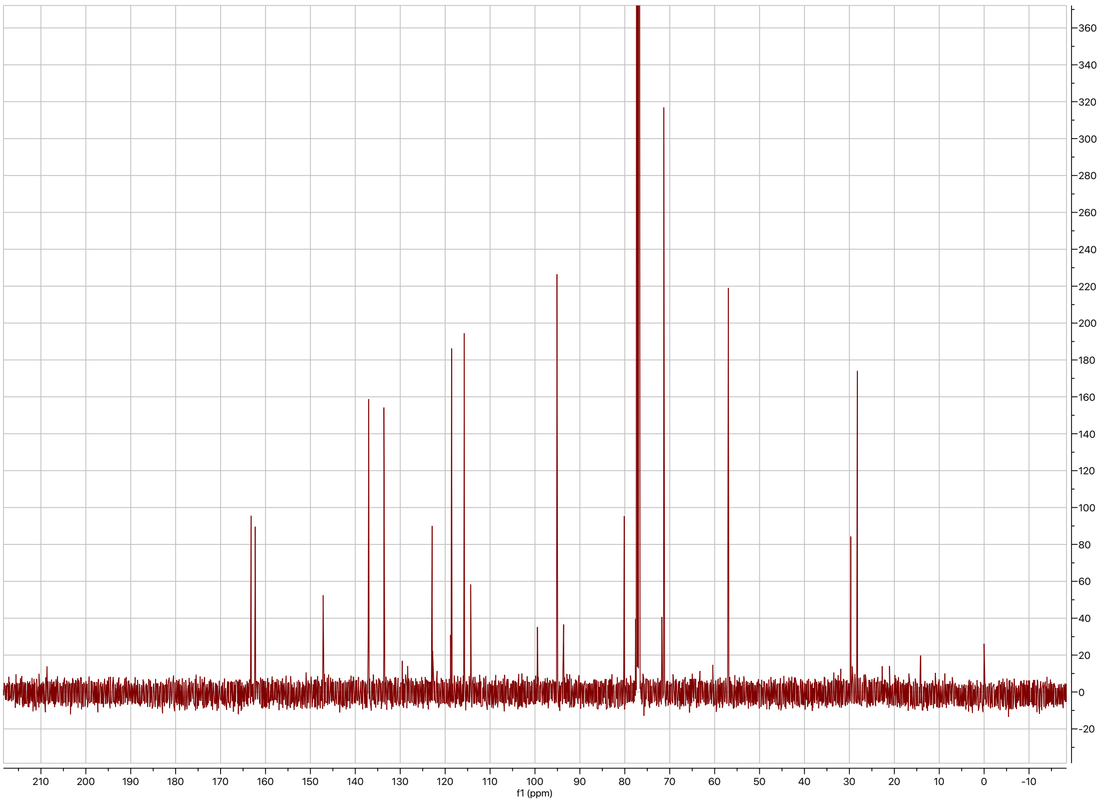

*1-(8-cyano-7-(methoxymethoxy)quinolin-2-yl)but-3-yn-1-yl-(2-(2-chloro-N-methylacetamido)ethyl)(methyl)carbamate (**3**).*

^{1}H NMR (500 MHz, chloroform-*d*)

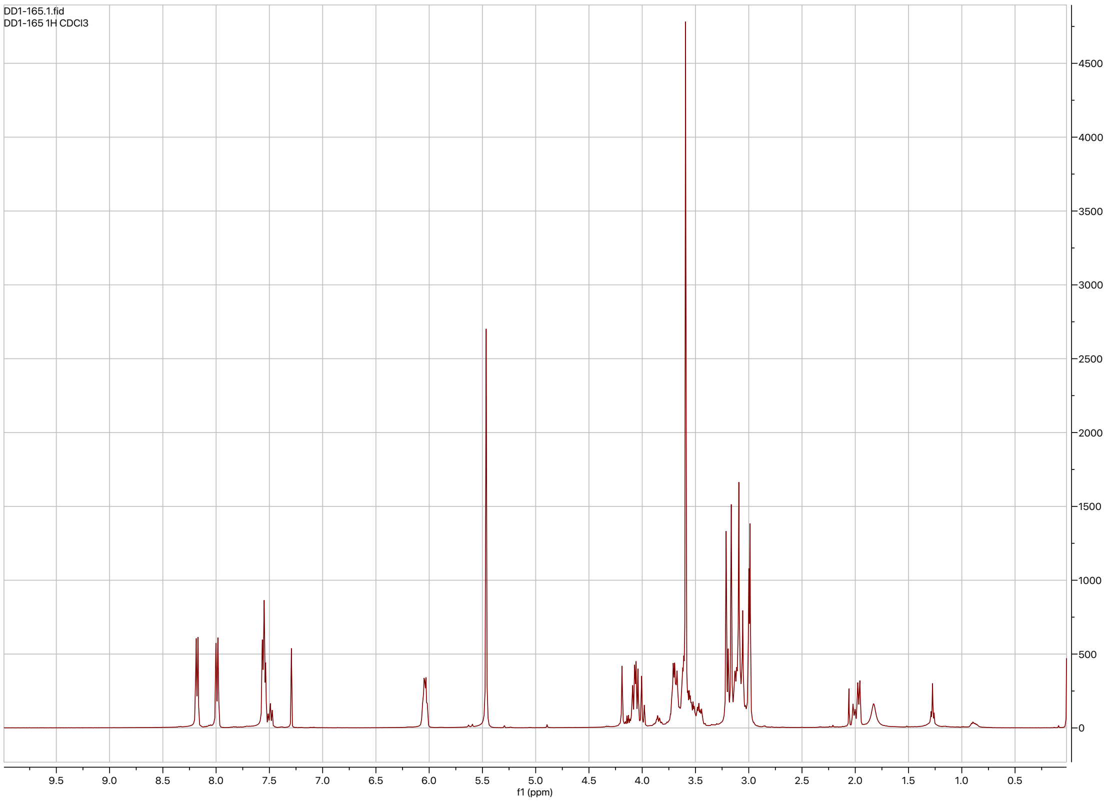

^{13}C NMR (126 MHz, chloroform-*d*)

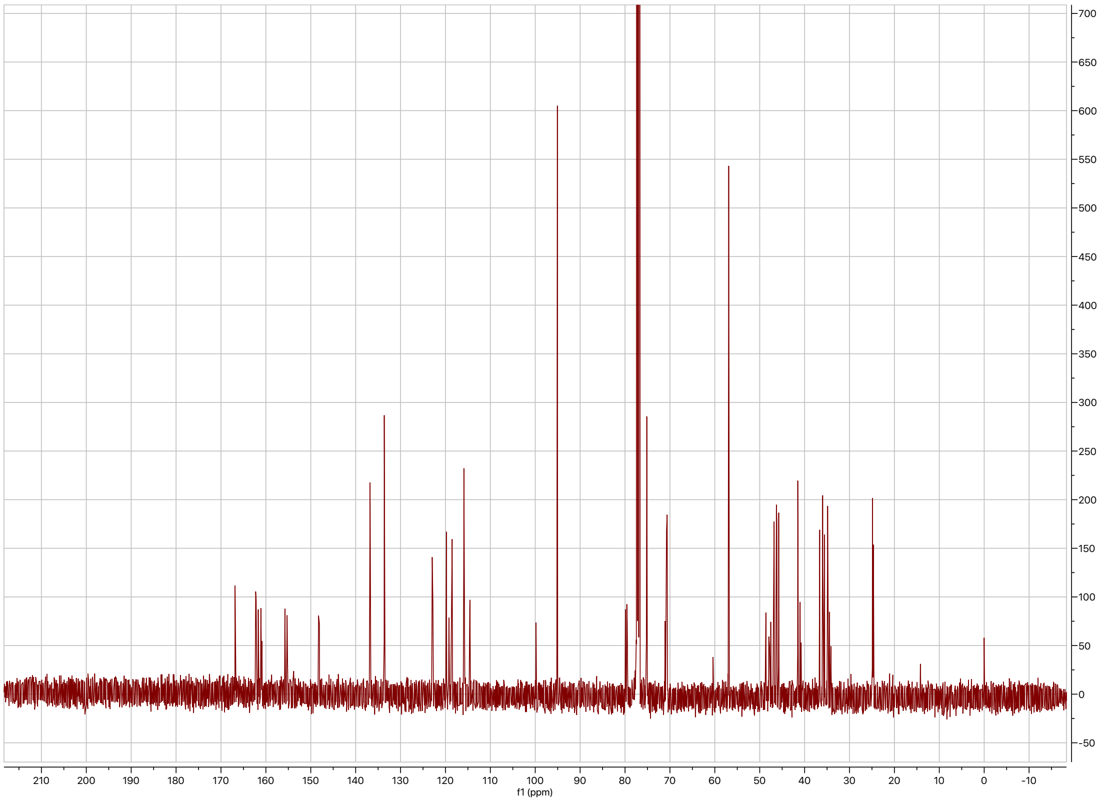

*1-(8-cyano-7-hydroxyquinolin-2-yl)but-3-yn-1-yl-(2-(2-chloro-N-methylacetamido)ethyl)(methyl)carbamate (**4**).*

^{1}H NMR (500 MHz, chloroform-*d*)

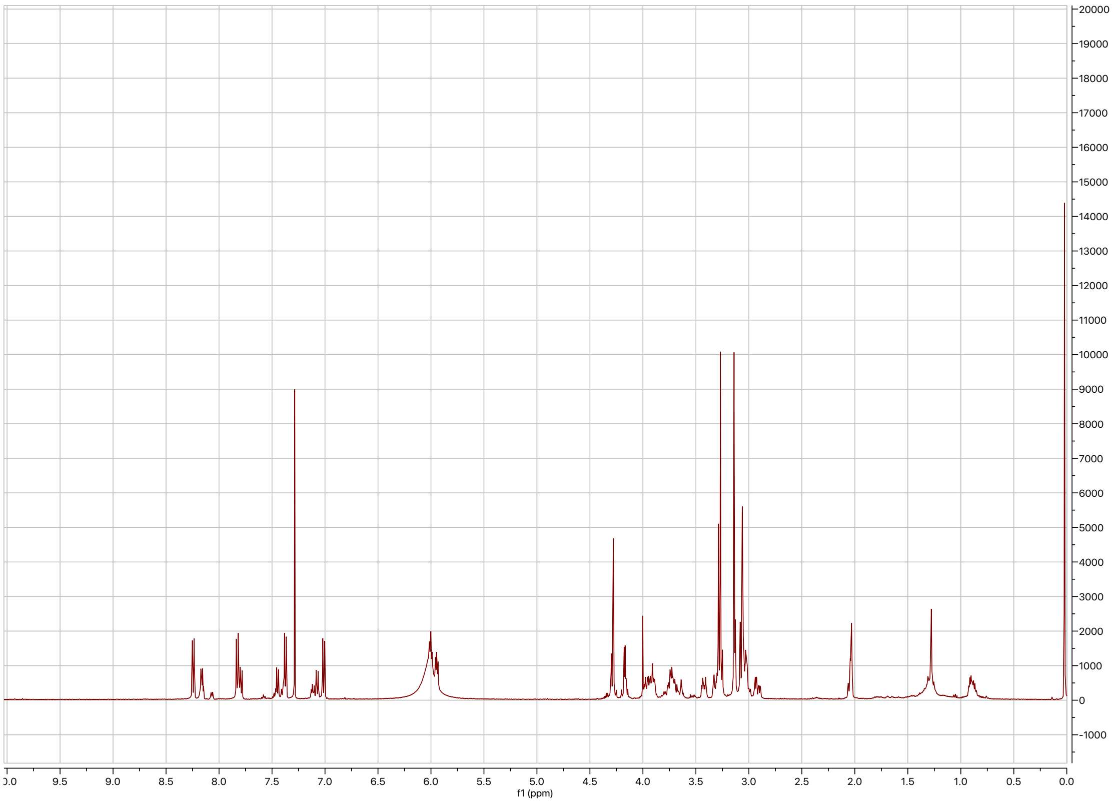

^{13}C NMR (126 MHz, chloroform-*d*)

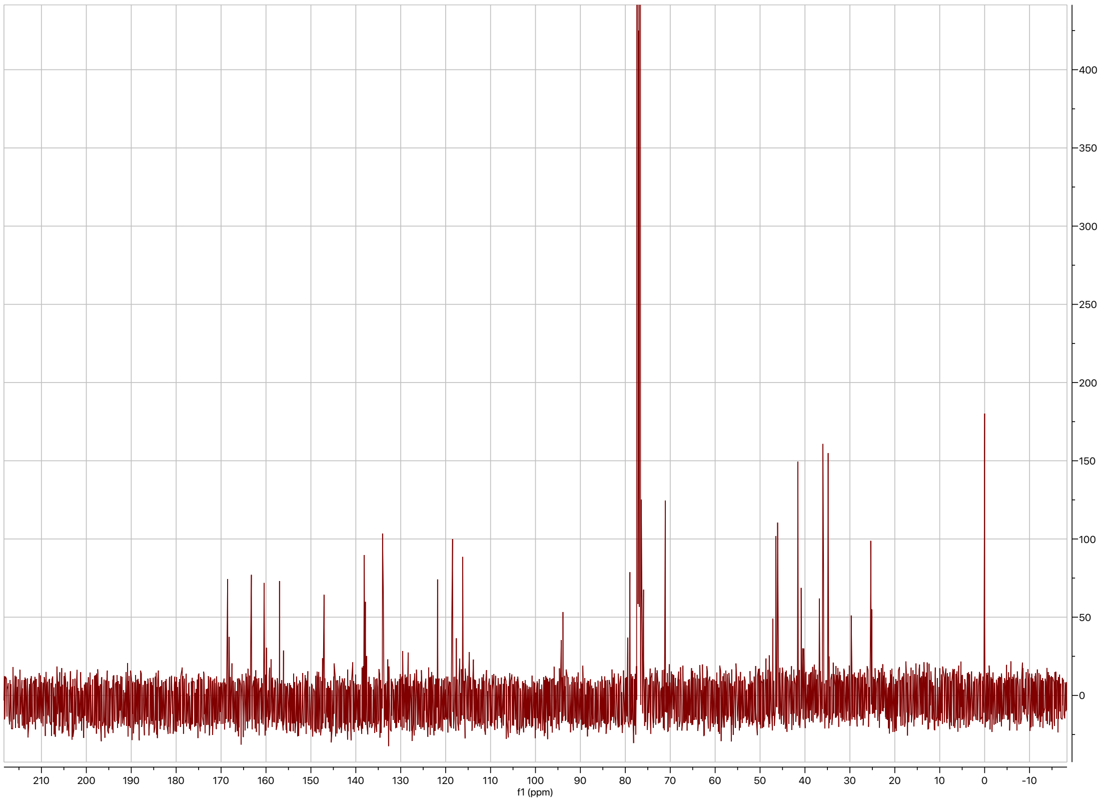

HRMS Spectra

*CyHQ-MO-gad1 (**6a**)*

[M] calculated for $C_{338}H_{515}N_{167}O_{103}S_2P_{25}Cl$ = 9439.7029

HRMS trace

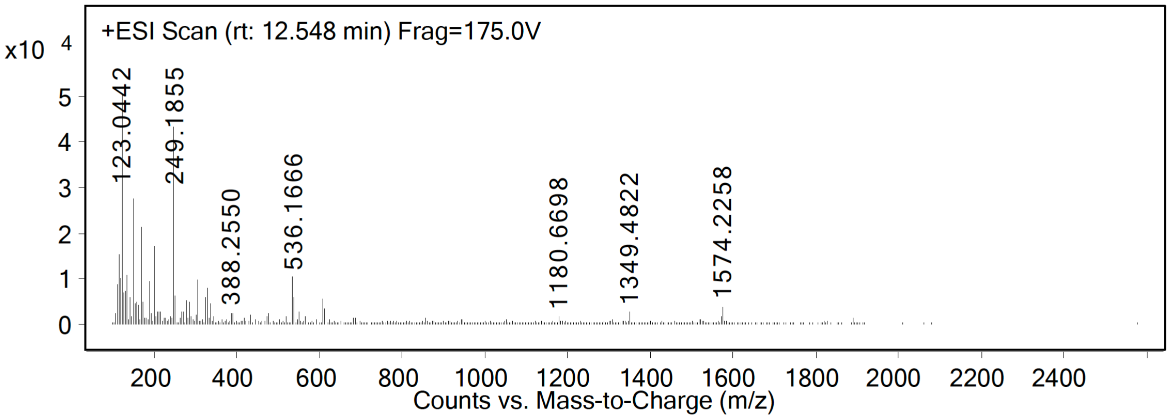

Deconvolution

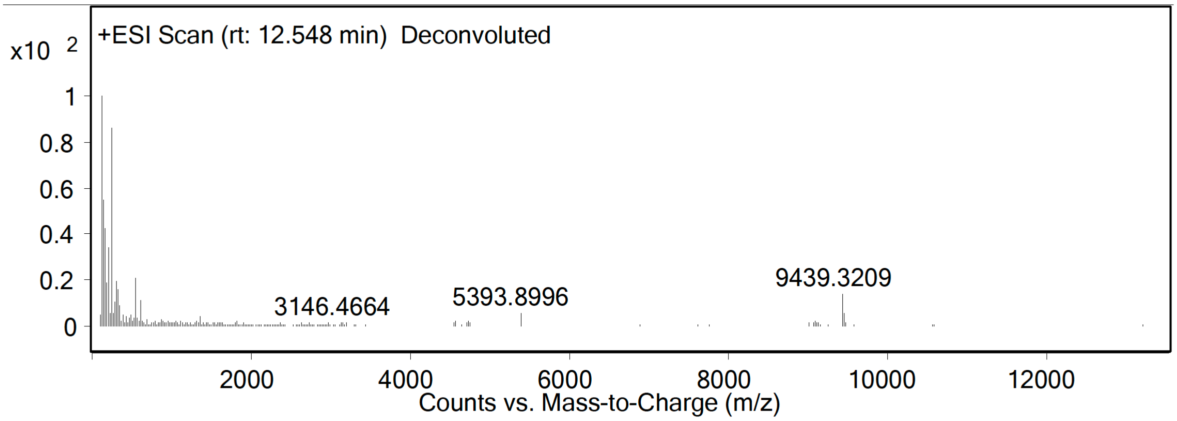

*CyHQ-MO-gad2 (**6b**)*

[M] calculated for $C_{338}H_{515}N_{164}O_{103}S_2P_{25}Cl$ = 9397.6828

HRMS trace

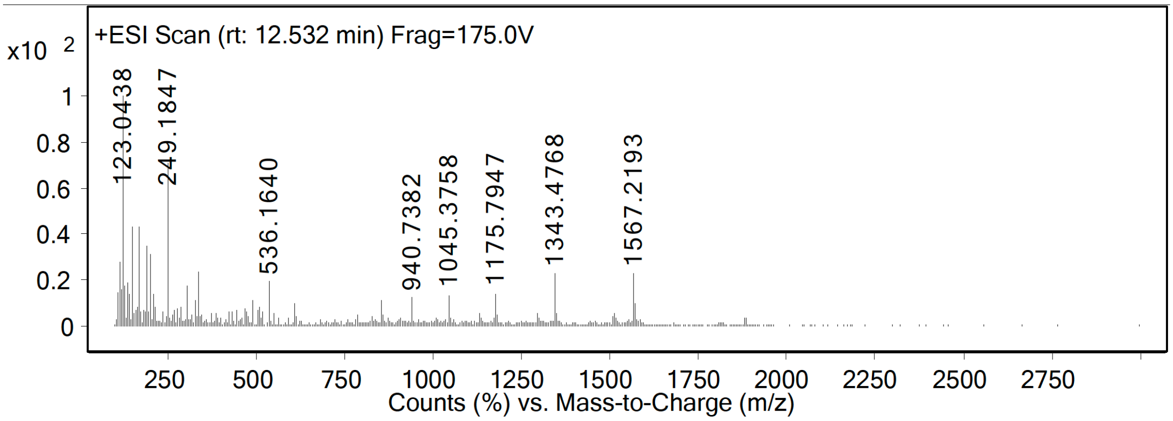

Deconvolution

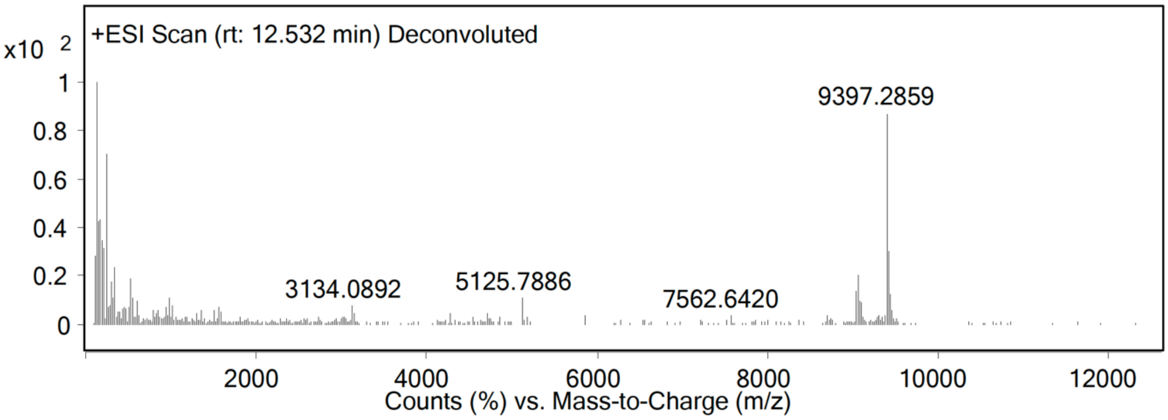

*CyHQ-ccMO-gad1 (**7a**)*

[M] calculated for $C_{334}H_{507}N_{166}O_{102}SP_{25}$ = 9285.0646

HRMS trace

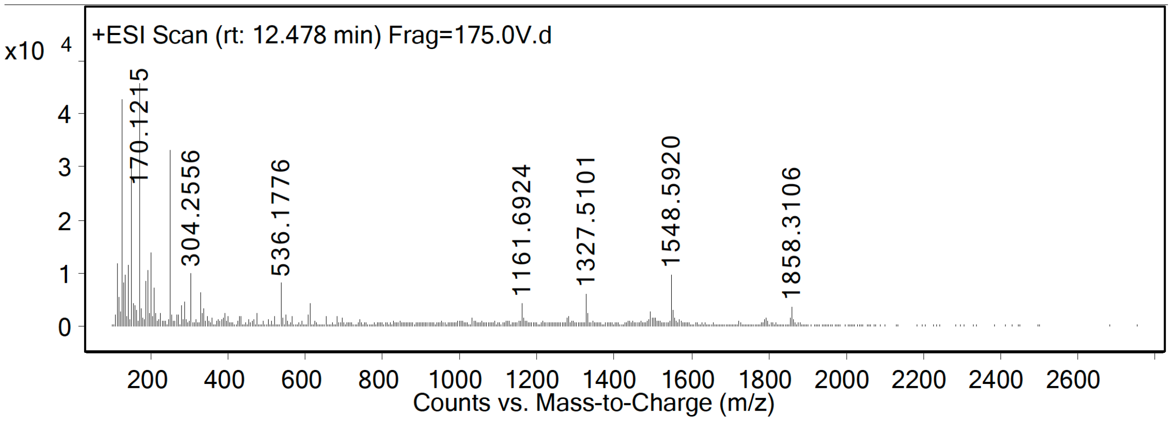

Deconvolution

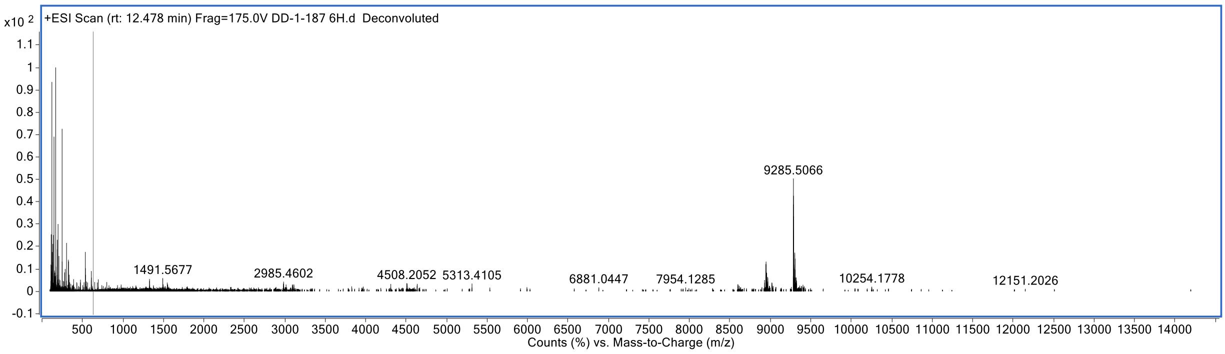

*CyHQ-ccMO-gad2 (**7b**)*

[M] calculated for $C_{334}H_{507}N_{163}O_{102}SP_{25}$ = 9244.0525

HRMS trace

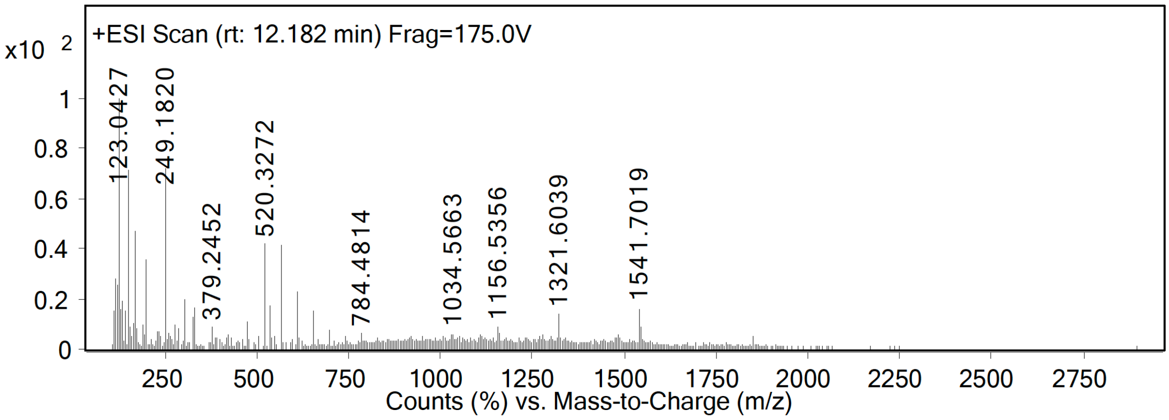

Deconvolution

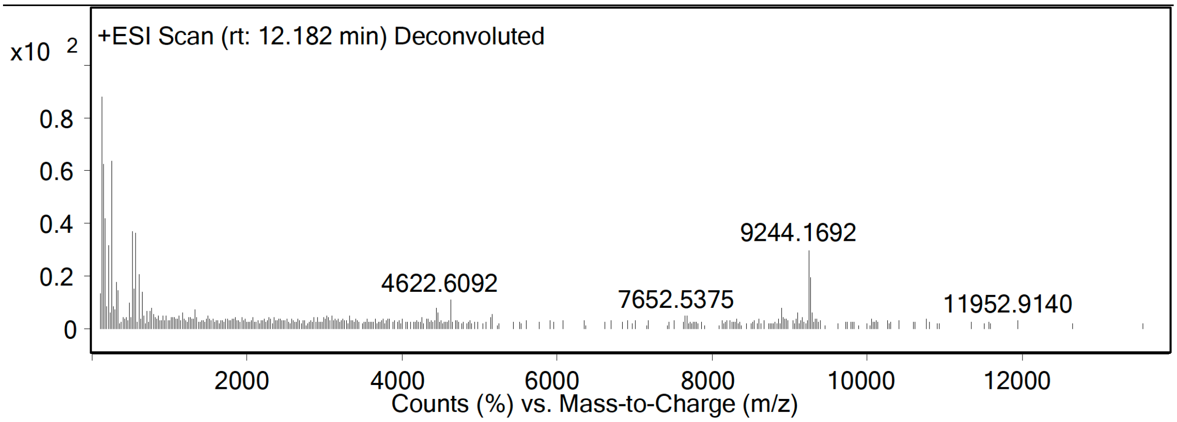

Supplement: Supplementary file 1 [file molecules-25-02078-s001.pdf]
